# Optimizing orchard management: a comparative study of single and dual-leader training systems in peach cultivation

**DOI:** 10.7717/peerj.21816

**Published:** 2026-11-12

**Authors:** Raşit Batur Oran, Dilan Ahı Koşar, Mevlüt Batuhan Koşar, Eküle Sönmez, Ümran Ertürk

**Affiliations:** 1 Plant Breeding Department, Viveros Provedo S.A., Logroño, La Rioja, Spain; 2 Department of Horticulture, Faculty of Agriculture, Bursa Uludag University, Bursa, Turkey; 3 Park and Horticulture, Landscape and Ornamental Cultivation Programme, Orhangazi Yeniköy Asil Çelik Vocational School, Bursa Uludag University, Bursa, Turkey

**Keywords:** Canopy architecture, Fruit quality, Labor efficiency, Peach, Training system, Yield efficiency

## Abstract

Rising labor costs and labor shortages are major constraints to sustainable peach production, particularly in traditional low-density orchards that rely on labor-intensive canopy architectures. Although high-density and simplified training systems are increasingly adopted worldwide, peach orchards in Türkiye remain largely based on conventional systems, limiting productivity and labor efficiency. This study evaluated two single-leader (Central Leader and Slender Spindle) and two dual-leader (Perpendicular-V and Tatura Trellis) training systems applied to three peach cultivars (Extreme^®^ 314, Extreme^®^ 436, and Extreme^®^ 568) under commercial conditions. During the study period, tree growth, labor requirements, yield performance, and fruit quality attributes were assessed. Central Leader produced greater vegetative vigor and canopy volume and generally high cumulative yield per tree, but was associated with greater thinning and harvesting labor. Slender Spindle maintained a compact canopy and showed the highest overall labor efficiency, whereas Perpendicular-V consistently had the lowest harvest labor per hectare. The highest cumulative yield per hectare was obtained with Slender Spindle in Extreme^®^ 314, Central Leader in Extreme^®^ 436, and Slender Spindle in Extreme^®^ 568. Tatura Trellis produced numerically higher cumulative yield per hectare than Perpendicular-V in all three cultivars, but also required greater cumulative labor. Fruit-quality responses varied among cultivar × training-system combinations. Overall, the relative performance of the training systems depended on cultivar and on whether yield, fruit quality, or labor requirements were prioritized.

## Introduction

Rising labor costs and labor shortages have become major challenges in fruit production, particularly in labor-intensive crops such as peach (Anthony & Minas, 2021; Lang, 2022; Neri et al., 2022). Traditional open vase systems, which dominate peach orchards worldwide, require extensive manual operations due to their large three-dimensional canopies, resulting in high pruning, thinning, and harvesting costs (Grossman & DeJong, 1998; Anthony & Minas, 2021). In Mediterranean production regions, labor expenses may account for up to 45% of total production costs, with harvesting, pruning, and thinning representing the largest shares (Foschi, Neri & Massetani, 2012).

To address these challenges, modern training systems have been developed to reduce labor requirements by controlling tree size, improving light interception, and facilitating cultural practices. These systems aim to produce smaller trees through increased planting density, simplified canopy architecture, and targeted pruning strategies, thereby enhancing orchard efficiency and fruit quality (Grossman & DeJong, 1998; Iglesias & Echeverria, 2022). However, because most peach rootstocks are not fully dwarfing, vegetative vigor must still be regulated through training system design and annual renewal pruning (Mazzoni et al., 2022). Peach trees naturally form sylleptic shoots with relatively wide branch angles, which, when combined with appropriate training systems, allow for the development of more open and manageable canopies (Neri, Massetani & Murri, 2015). Accordingly, peach training systems range from traditional three-dimensional architectures suitable for low- to medium-density plantings—such as Open Vase, Delayed Vase, Catalan Vase, Hex-V, and Quad-V—to modern planar or semi-planar systems designed for high-density orchards, including Palmette/hedge-row, Y-shaped systems, Fusetto, and Slender Spindle (Hoying, Robinson & Anderson, 2007; Minas, Tanou & Molassiotis, 2018; Mazzoni et al., 2022).

High-density planting systems (approximately 1,000–2,000 trees ha^−1^) maximize land use efficiency and improve canopy light distribution, which has been associated with enhanced soluble solids concentration, fruit coloration, and overall fruit quality (Anthony & Minas, 2021). These systems can be established either through central-axis architectures, such as Slender Spindle or Fusetto, or by forming inclined fruiting walls using dual-leader systems along tractor alleys (Loreti & Massai, 2006). The Tatura Trellis system was first developed in Australia (Chalmers, Mitchell & Van Heek, 1981) and subsequently followed by several Y-shaped system variants differing in planting density and branch angles. One of these, the Perpendicular-V system, was developed at University of California, Davis (DeJong et al., 1994). While similar in structure, the Perpendicular-V system allows higher tree density per unit area and facilitates pruning and thinning operations without requiring specialized labor. In contrast, central leader or central-axis systems, often combined with semi-dwarfing rootstocks, remain the dominant training approach in many European peach-producing countries, including Italy, Greece, and Spain, where they aim to form a conical canopy architecture broader at the base (Minas, Tanou & Molassiotis, 2018).

Despite being among the world’s leading peach producers, Türkiye continues to rely predominantly on traditional open vase systems characterized by low planting density and high labor demand. Limited adoption of modern training systems, increasing labor shortages, uneven fruit quality, and declining profitability highlight the need for alternative orchard designs adapted to local conditions. Moreover, cultivar-specific growth habits and fruiting behavior strongly influence the suitability of training systems, emphasizing the importance of evaluating cultivar × training system interactions rather than training systems alone (Iglesias & Echeverría, 2009; Sutton et al., 2020). Therefore, the objective of this study was to compare single-leader (Central Leader and Slender Spindle) and dual-leader (Perpendicular-V and Tatura Trellis) training systems applied to three newly released peach cultivars (Extreme^®^ 314, Extreme^®^ 436, and Extreme^®^ 568) in terms of tree growth, labor requirements, yield performance, fruit quality, and overall orchard efficiency under Turkish growing conditions.

## Materials and Methods

### Experimental site and plant material

The experiment was conducted between 2018 and 2022 in a commercial peach orchard located in Bursa Province, Türkiye (40°11′N, 29°04′E; 155 m a.s.l.), characterized by a temperate climate with cold winters and warm summers. The soil at the experimental site was classified as clay–loam, with good drainage and moderate organic matter content. Standard cultural practices, including fertilization, irrigation, pest, and disease management, were applied uniformly across all treatments according to regional recommendations.

Three newly released peach cultivars—Extreme^®^ 314 (early-season), Extreme^®^ 436 (mid-season), and Extreme^®^ 568 (late-season)—grafted onto GxN15 (Garnem) (Garfi × Nemared) (Felipe, 2009) rootstock were used in this study. Trees were planted in the winter of 2017 and trained to four different leader-based training systems: Central Leader (CL), Slender Spindle (SS), Perpendicular-V (PV), and Tatura Trellis (TT). Row spacing was fixed at 5.0 m for all systems, while in-row spacing varied according to training system: 2.5 m for CL, approximately 1.27 m for PV and TT, and 1.0 m for SS, corresponding to planting densities of 800, 1580, 1580, and 2000 trees ha^−1^, respectively.

### Training systems and orchard management

In the CL system, trees were trained to a single vertical axis with scaffold branches arranged spirally along the trunk. The SS system also consisted of a single leader but emphasized narrow canopy width and high planting density, with limited branch diameter maintained through annual renewal pruning. The PV and TT systems were trained with two primary leaders inclined at approximately 35–40° from the vertical in PV and 70–80° in TT. In the TT system, trees were supported by a permanent trellis structure consisting of galvanized steel posts and wires to maintain branch orientation, whereas no support structure was used in the PV system. Training and pruning practices were applied annually to maintain the intended canopy architecture in each system.

### Phenological evaluation

Phenological development was observed during each growing season. Flowering period was defined as the interval between 10% bloom and petal fall, while fruit development duration was calculated as the number of days from full bloom to harvest. The harvest period was recorded as the total number of days required to complete fruit harvest for each treatment and cultivar.

### Tree growth measurements

Tree height was measured annually at the end of the growing season using a telescopic measuring pole (m). Trunk cross-sectional area (TCSA) was calculated from trunk diameter measured 30 cm above the graft union using a digital caliper (Mitutoyo Corp. Kawasaki, Japan), applying the formula TCSA = *π*r^2^. Canopy volume was estimated according to the geometric method described by Robinson, Andersen & Hoying (2006), considering tree height and average canopy width. Summer and winter pruning weights were recorded separately for each tree using a digital scale (±0.01 kg; Kern & Sohn GmbH, Balingen-Frommern, Germany). Pruning labor time was recorded during each pruning operation and expressed as minutes per tree, then converted to hours per hectare.

### Yield and yield components

Fruit yield was recorded at each harvest and expressed on both a per-tree (kg tree^−1^) and per-hectare (t ha^−1^) basis. Cumulative yield was calculated as the sum of annual yields over the experimental period. Annual yield efficiency was calculated as annual yield per tree divided by the corresponding annual TCSA. Cumulative yield efficiency (CYE, kg cm^−2^) was calculated as the sum of annual yield efficiency values for 2018, 2019, 2020, and 2022. Annual crop load was calculated as the number of fruits per tree divided by the corresponding annual TCSA. Cumulative crop load (CCL, fruits cm^−2^) was calculated as the sum of annual crop load values for 2018, 2019, 2020, and 2022. Harvest was conducted manually and completed in two to three passes, depending on cultivar and training system, based on commercial maturity criteria. Yield precocity was assessed as the yield obtained during the first productive year.

### Fruit quality measurements

At each harvest, representative fruit samples were collected to assess fruit quality attributes. Fruit weight was determined using a digital balance (±0.1 g). Flesh firmness was measured on two opposite peeled sides of each fruit using a digital penetrometer equipped with an 8-mm probe (FT327, Effegi, Brembate Sopra, Italy) and expressed as kg cm^−2^. Soluble solids content (SSC) was measured using a digital refractometer (Atago Co. Ltd., Tokyo, Japan) and expressed as ^∘^Brix. Titratable acidity (TA) was determined by titration with 0.1 N NaOH and expressed as g malic acid per 100 mL of juice. Skin and flesh color were measured using a portable colorimeter (Minolta CR-400, Konica Minolta, Tokyo, Japan), and color parameters were expressed as hue angle (^∘^).

### Labor assessment

Labor requirements for pruning (summer and winter), thinning, harvesting, and other cultural practices were recorded annually. Labor time was expressed as hours per tree and converted to hours per hectare (h ha^−1^). Labor efficiency was calculated as fruit yield per unit labor input (kg h^−1^).

### Experimental design and statistical analysis

The experiment was arranged in a randomized complete block design with four replications, each consisting of five trees per treatment. Measurements were collected from all trees within each plot, and the mean value of the five trees was used as the experimental unit for statistical analyses. For cumulative and final response variables, data were subjected to analysis of variance (ANOVA) using a model that included training system, cultivar, training system × cultivar interaction, and block (replication). Training system and cultivar were treated as fixed factors, while block was included in the model to account for variation among the four replications. Mean separations were performed using Tukey’s test when significant effects were observed (*P* ≤ 0.05). Annual yield data were analyzed separately for each cultivar and year; within each cultivar × year combination, training systems were compared using the four blocks, with block included in the model. Because years were analyzed independently rather than jointly across seasons, year was not included as a repeated factor and no across-year covariance structure was specified. In 2021, severe frost resulted in extremely low fruit availability; therefore, yield was not measured and was not treated as zero. Cumulative yield was consequently calculated from the measured yield seasons (2018, 2019, 2020, and 2022). Pruning operations were performed and recorded in 2021 and were included in cumulative pruning labor, whereas thinning and harvesting were not performed because of insufficient fruit availability. ANOVA was conducted using JMP Pro 16 (SAS Institute Inc., Cary, NC, USA), and complete effect-test summaries (df, F, and *P* values) are provided in Table S1. Pearson correlation analyses were performed separately for each cultivar using plot-level observations (four training systems × four blocks; *n* = 16 per cultivar) and were interpreted as exploratory. Principal component analysis (PCA) was performed using the 48 plot-level observations after standardization, whereas the heatmap was constructed from the 12 cultivar × training-system treatment means. PCA, correlation matrices, and heatmap visualization were performed using OriginPro 2024 (OriginLab Corporation, Northampton, MA, USA) as exploratory multivariate summaries.

## Results

### Phenological evaluation

Training systems induced modest but consistent differences in flowering and harvest timing, while cultivar-related effects were more pronounced. The TT and PV systems extended the flowering period of Extreme^®^ 314 by approximately two days (14 days) compared with CL and SS systems, whereas Extreme^®^ 568 consistently exhibited the longest fruit development duration regardless of training system (Fig. 1). Earlier ripening and a shorter harvest (1–2 days) were generally observed in trees trained to SS and CL systems for all cultivars.

**Figure 1:**
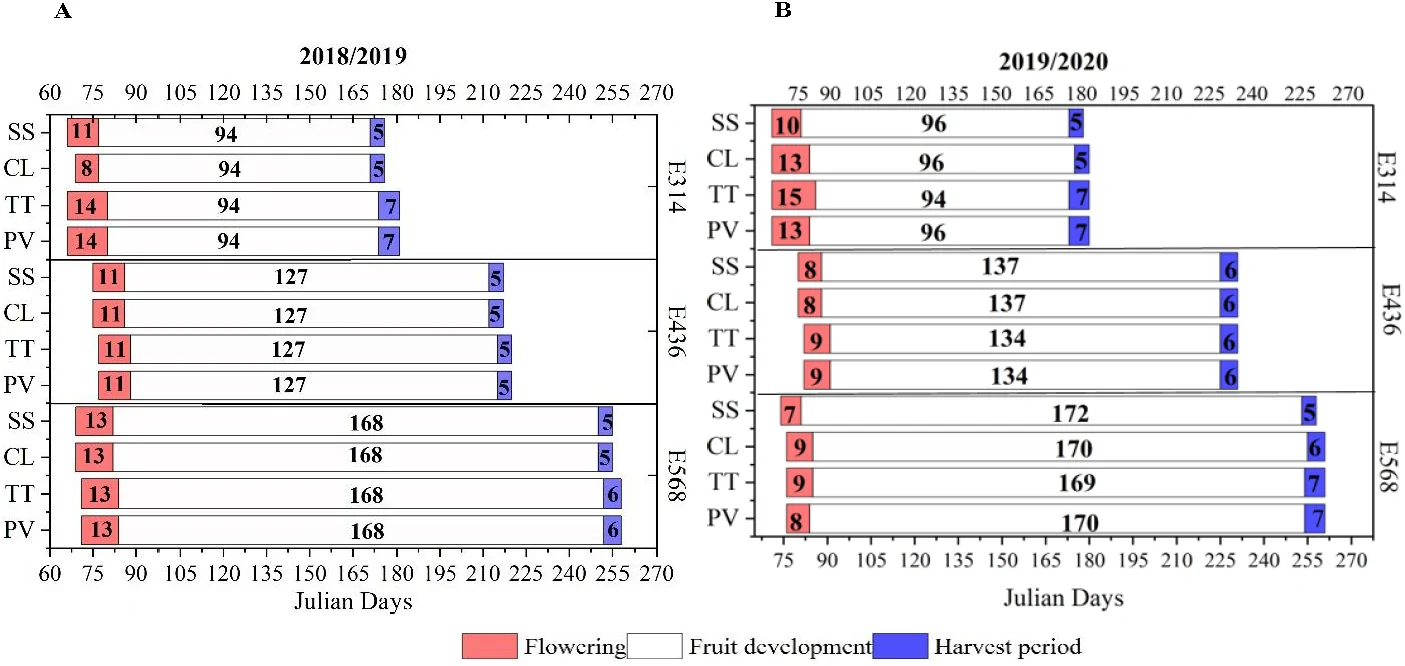
Flowering period, fruit development, and harvest period of peach cultivars Extreme^®^ 314 (E314), Extreme^®^ 436 (E436), and Extreme^®^ 568 (E568) according to different training systems in 2018/2019 (A) and 2019/2020 production cycles.

### Tree growth characteristics

The training system significantly affected tree height, TCSA, and canopy volume (Table 1; Table S1), whereas the training system × cultivar interaction was not significant for these traits. Regardless of cultivar, the highest TCSA was recorded in the CL system, followed by the PV system. Extreme^®^ 314 (82.6 cm^2^) and Extreme^®^ 568 (73.6 cm^2^) exhibited higher TCSA values than Extreme^®^ 436 (69.6 cm^2^). As with TCSA, the highest canopy volume was observed in the CL system, while the SS system consistently resulted in the lowest canopy volume across cultivars (Table 1).

The training system also significantly affected cumulative summer and winter pruning weights (Table 2; Table S1). A significant training system × cultivar interaction was detected for winter pruning weight (Table S1). The highest summer and winter pruning weights were recorded in the CL system for all cultivars, while winter pruning weights did not differ significantly among cultivars. Higher summer pruning weights were observed in Extreme^®^ 568 (4.8 kg tree^−1^) and Extreme^®^ 314 (4.4 kg tree^−1^) (Table 2).

**Table 1:** Tree growth characteristics and labor analysis of peach cultivars trained to different training systems. Values are presented as mean ± SE (*n* = 4).

Cultivars	Training system	Final			Cumulative									
		TH (cm)	TCSA (cm^2^)	TCV (m^3^)	SPW	WPW	SPL^i^		WPL^i^		TL^ii^		HL^ii^	
					kg tree^−1^	kg tree^−1^	min tree^−1^	h ha^−1^	min tree^−1^	h ha^−1^	min tree^−1^	h ha^−1^	min tree^−1^	h ha^−1^
Extreme^®^ 314	PV	301.7 ± 8.8 b	83.7 ± 5.8 a^iii^	12.6 ± 1.0 b	4.4 ± 0.48 b	12.6 ± 0.92 ab	25.2 ± 1.5 b	664.7 ± 38.2 ab	28.4 ± 0.64 b	749.0 ± 16.9 bc	13.2 ± 0.29 b	351.4 ± 7.7 b	5.0 ± 0.20 c	132.6 ± 5.2 c
	TT	302.0 ± 10.8 b	92.8 ± 3.1 a	12.5 ± 1.2 b	4.3 ± 0.62 b	12.9 ± 1.3 ab	27.0 ± 0.43 b	711.0 ± 11.4 a	32.1 ± 0.92 b	847.6 ± 24.3 a	13.3 ± 0.19 b	348.5 ± 4.9 b	7.6 ± 0.14 b	200.5 ± 3.6 b
	CL	311.0 ± 9.4 ab	96.8 ± 6.8 a	27.4 ± 1.7 a	6.5 ± 0.55 a	14.8 ± 0.38 a	39.9 ± 0.46 a	532.0 ± 6.1 bc	61.5 ± 1.6 a	820.4 ± 21.2 ab	25.1 ± 0.90 a	335.5 ± 12.0 b	20.5 ± 0.16 a	273.6 ± 2.1 a
	SS	328.9 ± 11.4 a	57.3 ± 6.4 b	5.5 ± 0.33 c	2.7 ± 0.51 c	10.5 ± 0.82 b	17.5 ± 1.1 c	586.1 ± 35.1 c	21.7 ± 1.2 c	725.0 ± 39.4 c	11.8 ± 0.29 b	394.7 ± 9.7 a	5.5 ± 0.20 c	184.6 ± 6.5 b
	Average	310.9	82.6 A	14.5	4.4 AB	12.7	27.4	623.4	35.9	785.5	15.8 B	357.5 B	9.7 B	197.8 B
Extreme^®^ 436	PV	285.6 ± 16.4 c	75.6 ± 7.8 ab	11.4 ± 0.83 b	4.0 ± 0.43 b	10.7 ± 1.4 b	24.3 ± 1.2 b	642.2 ± 31.5 ab	29.5 ± 1.3 b	776.3 ± 33.1 b	14.9 ± 0.34 bc	393.7 ± 8.9 c	7.4 ± 0.14 c	195.4 ± 3.7 c
	TT	302.1 ± 10.9 bc	66.3 ± 3.3 bc	11.7 ± 0.65 b	3.3 ± 0.50 bc	10.3 ± 0.94 b	28.9 ± 0.53 b	761.0 ± 13.9 a	33.0 ± 1.3 b	869.0 ± 34.0	16.5 ± 0.29 b	435.2 ± 7.6 bc	9.0 ± 0.25 bc	236.4 ± 6.6 bc
	CL	333.7 ± 12.7 ab	88.6 ± 9.6 a	29.5 ± 2.8 a	5.7 ± 0.51 a	17.6 ± 1.8 a	40.7 ± 0.75 a	543.3 ± 10.0 b	61.0 ± 1.3 a	813.3 ± 17.2	44.6 ± 1.6 a	595.3 ± 21.7 a	41.8 ± 1.0 a	556.9 ± 14.0 a
	SS	337.8 ± 4.1 a	47.9 ± 3.3 c	5.8 ± 0.35 c	2.9 ± 0.18 c	12.1 ± 0.75 b	18.0 ± 1.4 c	601.0 ± 47.3 b	23.0 ± 1.5 c	768.3 ± 48.3 b	13.4 ± 0.34 c	448.4 ± 11.3 b	7.9 ± 0.14 b	264.1 ± 4.6 b
	Average	314.8	69.6 B	14.6	3.9 B	12.6	27.9	636.8	36.6	806.7	22.3 A	468.2 A	16.5 A	313.2 A
Extreme^®^ 568	PV	296.6 ± 8.8 ab	82.0 ± 10.1 a	11.5 ± 0.90 b	5.0 ± 0.35 b	11.8 ± 1.3 b	23.2 ± 1.7 bc	612.2 ± 43.5 ab	28.7 ± 1.2 b	757.0 ± 32.9	12.4 ± 0.53 bc	328.2 ± 14.0 c	4.6 ± 0.04 c	123.5 ± 1.1 c
	TT	290.8 ± 7.2 b	72.4 ± 6.5 ab	11.5 ± 0.92 b	4.1 ± 0.69 b	10.5 ± 0.52 b	28.0 ± 1.1 b	738.6 ± 28.9 a	28.8 ± 2.2 b	759.7 ± 58.8	15.9 ± 0.41 b	420.9 ± 10.9 b	6.2 ± 0.14 b	164.8 ± 3.7 b
	CL	317.4 ± 10.0 a	85.2 ± 7.3 a	24.5 ± 1.4 a	6.4 ± 0.71 a	15.1 ± 0.78 a	39.5 ± 1.0 a	526.6 ± 13.9 b	62.5 ± 1.7 a	833.3 ± 22.1	35.8 ± 1.5 a	477.6 ± 20.1 a	17.1 ± 0.09 a	229.1 ± 1.2 a
	SS	314.8 ± 7.4 a	54.9 ± 5.3 b	4.8 ± 0.26 c	4.0 ± 0.08 b	14.8 ± 0.32 a	19.3 ± 1.3 c	645.5 ± 43.8 ab	22.4 ± 1.6 c	748.6 ± 52.3	10.9 ± 0.53 c	365.5 ± 17.7 c	5.1 ± 0.04 c	173.0 ± 1.4 b
	Average	304.9	73.6 AB	13.0	4.8 A	13.0	27.5	630.7	35.6	774.6	18.7 AB	398.0 AB	8.2 B	172.6 B

**Notes.**

I Cumulative labor (2018–2021).

II Cumulative labor (2019–2022).

III Different lowercase letters indicate differences among training systems within each cultivar, while uppercase letters indicate differences among cultivars according to Tukey’s test (*P* ≤ 0.05).

PV, Perpendicular-V; TT, Tatura Trellis; CL, Central Leader; SS, Slender Spindle; TH, tree height; TCSA, trunk cross-sectional area; TCV, tree canopy volume; SPW, summer pruning weight; WPW, winter pruning weight; SPL, summer pruning labor; WPL, winter pruning labor; TL, thinning labor; HL, harvest labor.

**Table 2:** Yield, crop load and fruit characteristics of peach cultivars trained to different training systems. Values are presented as mean ± SE (*n* = 4).

Cultivar	Training system	CY (kg tree^−1^)	CY (ton ha^−1^)	CYE (kg cm^−2^ )	CCL (fruit number per cm^2^)	FW (g)	FF (kg/cm^2^ )	SSC (^∘^Brix)	TA (g/100 ml)	Skin Hue (^∘^)	Flesh Hue (^∘^)
Extreme^®^ 314	PV	12.3 ± 0.74 b	19.5 ± 1.2 b	0.33 ± 0.02 b	1.82 ± 0.19	164.7 ± 2.9	4.73 ± 0.12 ab	11.87 ± 0.32 b	0.36 ± 0.03	27.6 ± 1.1	89.6 ± 0.51
	TT	17.2 ± 1.1 b	27.2 ± 1.7 ab	0.38 ± 0.03 b	1.94 ± 0.23	167.1 ± 4.3	5.00 ± 0.12 a	12.62 ± 0.37 ab	0.33 ± 0.03	28.3 ± 1.3	89.8 ± 0.55
	CL	24.6 ± 2.8 a	19.7 ± 2.3 b	0.50 ± 0.09 ab	2.84 ± 0.68	165.3 ± 3.5	4.76 ± 0.08 ab	11.66 ± 0.20 b	0.36 ± 0.02	29.5 ± 1.8	91.4 ± 1.2
	SS	15.4 ± 2.0 b	30.8 ± 4.1 a	0.63 ± 0.12 a	2.74 ± 0.45	164.2 ± 4.8	4.39 ± 0.09 b	13.16 ± 0.14 a	0.34 ± 0.02	27.2 ± 0.87	90.2 ± 1.1
	Average	17.3 B	24.3 B	0.45 B	2.33 AB	165.3 C	4.72	12.27 C	0.34	28.15 B	90.25 A
Extreme^®^ 436	PV	18.0 ± 1.9 b	28.4 ± 3.0 b	0.49 ± 0.10 b	1.75 ± 0.31 b	200.7 ± 2.5 ab	5.94 ± 0.19 a	15.85 ± 0.35	0.39 ± 0.02 ab	34.5 ± 1.9	75.7 ± 1.7
	TT	21.6 ± 1.8 b	34.2 ± 2.8 ab	0.73 ± 0.03 ab	2.75 ± 0.20 b	193.6 ± 6.3 b	5.38 ± 0.26 ab	15.78 ± 0.37	0.37 ± 0.01 bc	36.7 ± 3.7	78.9 ± 1.5
	CL	54.2 ± 7.6 a	43.4 ± 6.1 a	1.33 ± 0.23 a	6.69 ± 1.19 a	179.5 ± 6.2 c	4.95 ± 0.13 b	15.86 ± 0.26	0.33 ± 0.02 c	33.9 ± 1.2	76.9 ± 1.3
	SS	16.9 ± 0.58 b	33.9 ± 1.2 ab	0.76 ± 0.04 ab	2.23 ± 0.13 b	211.7 ± 3.5 a	4.93 ± 0.23 b	15.55 ± 0.16	0.43 ± 0.03 a	35.5 ± 1.3	79.7 ± 1.3
	Average	27.7 A	35.0 A	0.82 A	3.32 A	196.3 B	5.30	15.70 A	0.38	35.15 A	77.80 B
Extreme^®^ 568	PV	11.7 ± 1.1 c	18.4 ± 1.8 b	0.34 ± 0.02 c	1.12 ± 0.10 c	233.8 ± 7.1 ab	4.89 ± 0.31	14.87 ± 0.50	0.29 ± 0.01 b	39.2 ± 1.2 a	77.9 ± 0.47 b
	TT	18.2 ± 2.7 ab	28.8 ± 4.3 ab	0.47 ± 0.09 bc	1.72 ± 0.35 bc	240.1 ± 5.4 a	4.93 ± 0.15	14.77 ± 0.25	0.35 ± 0.02 a	34.5 ± 1.6 b	83.2 ± 0.79 a
	CL	23.9 ± 1.6 a	19.1 ± 1.3 b	0.67 ± 0.05 a	2.46 ± 0.20 a	247.2 ± 4.3 a	5.03 ± 0.36	14.35 ± 0.37	0.34 ± 0.01 a	34.5 ± 1.0 b	77.0 ± 2.0 b
	SS	15.4 ± 1.2 bc	30.9 ± 2.5 a	0.52 ± 0.03 ab	1.89 ± 0.12 ab	217.7 ± 5.1 b	5.23 ± 0.08	14.27 ± 0.04	0.34 ± 0.01 a	40.4 ± 0.58 a	80.2 ± 0.84 ab
	Average	17.3 B	24.3 B	0.50 B	1.79 B	234.7 A	5.02	14.50 B	0.33	37.15 A	79.57 B

**Notes.**

I Lowercase letters indicate differences among training systems within each cultivar, while uppercase letters indicate differences among cultivars according to Tukey’s test (*P* ≤ 0.05).

PV, Perpendicular-V; TT, Tatura Trellis; CL, Central Leader; SS, Slender Spindle; CY, cumulative yield; CYE, cumulative yield efficiency; CCL, cumulative crop load; FW, fruit weight; FF, fruit firmness; SSC, soluble solids content; TA, titratable acidity.

### Labor assessment

The training system significantly affected cumulative summer and winter pruning labor across all cultivars (Table 1; Table S1), whereas the training system × cultivar interactions for these two pruning-labor variables were not significant. Across cultivars, summer pruning labor per hectare was highest in TT, while winter pruning labor was similarly high in TT and CL. Training systems also significantly influenced thinning and harvesting labor, with significant training system × cultivar interactions for both variables (Table S1). Harvest labor per hectare was highest in CL and lowest in PV across all cultivars. Compared with CL, harvest labor per hectare was lower by approximately 57% in PV, 43% in TT, and 41% in SS. A significant training system × cultivar interaction was also detected for cumulative labor (Table S1); when averaged across cultivars, PV had the lowest cumulative labor, whereas TT and CL were among the highest. Labor efficiency was significantly affected by training system and cultivar, but the interaction was not significant. SS had the highest overall mean labor efficiency (Fig. 2).

**Figure 2:**
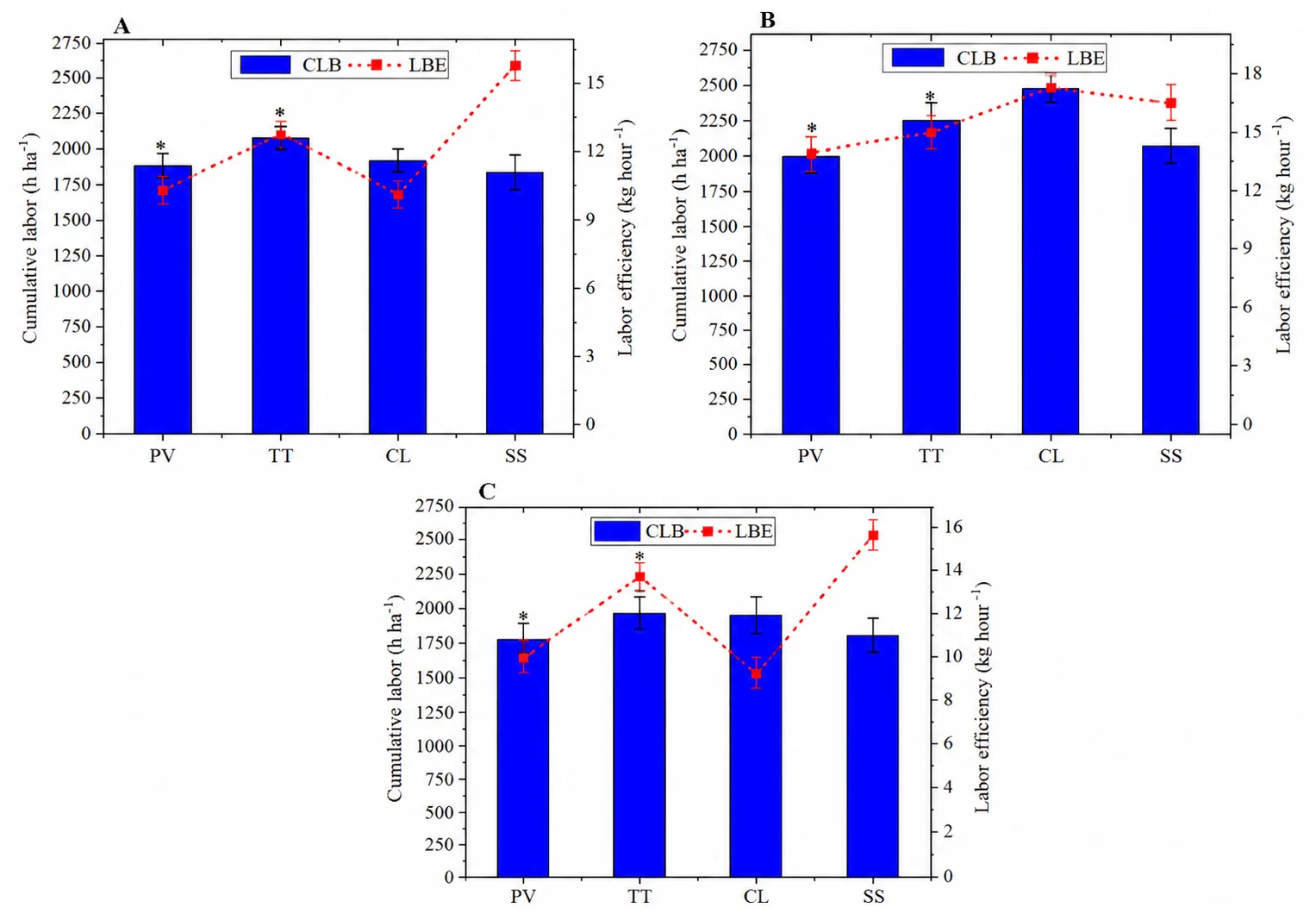
Cumulative labor (2019–2022) required for major cultural practices (h ha^−1^) and labor efficiency (kg ha^−1^) of Extreme^®^ 314 (A), Extreme^®^ 436 (B), and Extreme^®^ 568 (C) peach cultivars trained to different training systems. PV, Perpendicular-V; TT, Tatura Trellis; CL, Central Leader; SS, Slender Spindle. Values are presented as mean ±SE (*n* = 4). * indicates statistical significance at *P* ≤ 0.05.

### Yield, cumulative yield, and yield efficiency

Regarding yield precocity, all training systems initiated fruit production in the second leaf after planting. In the first year, the highest yield was obtained from trees trained to the SS system for Extreme^®^ 314 (0.79 kg tree^−1^) and Extreme^®^ 436 (1.76 kg tree^−1^), whereas Extreme^®^ 568 (1.40 kg tree^−1^) produced the highest yield under the TT system (Fig. 3). In subsequent measured years, the relative performance of the training systems varied among cultivar × year combinations and according to whether yield was expressed per tree or per hectare. In 2021, severe frost resulted in extremely low fruit availability; therefore, yield was not measured in that season. The significance of training-system effects within each cultivar × year combination is indicated by * or ns in Fig. 3.

**Figure 3:**
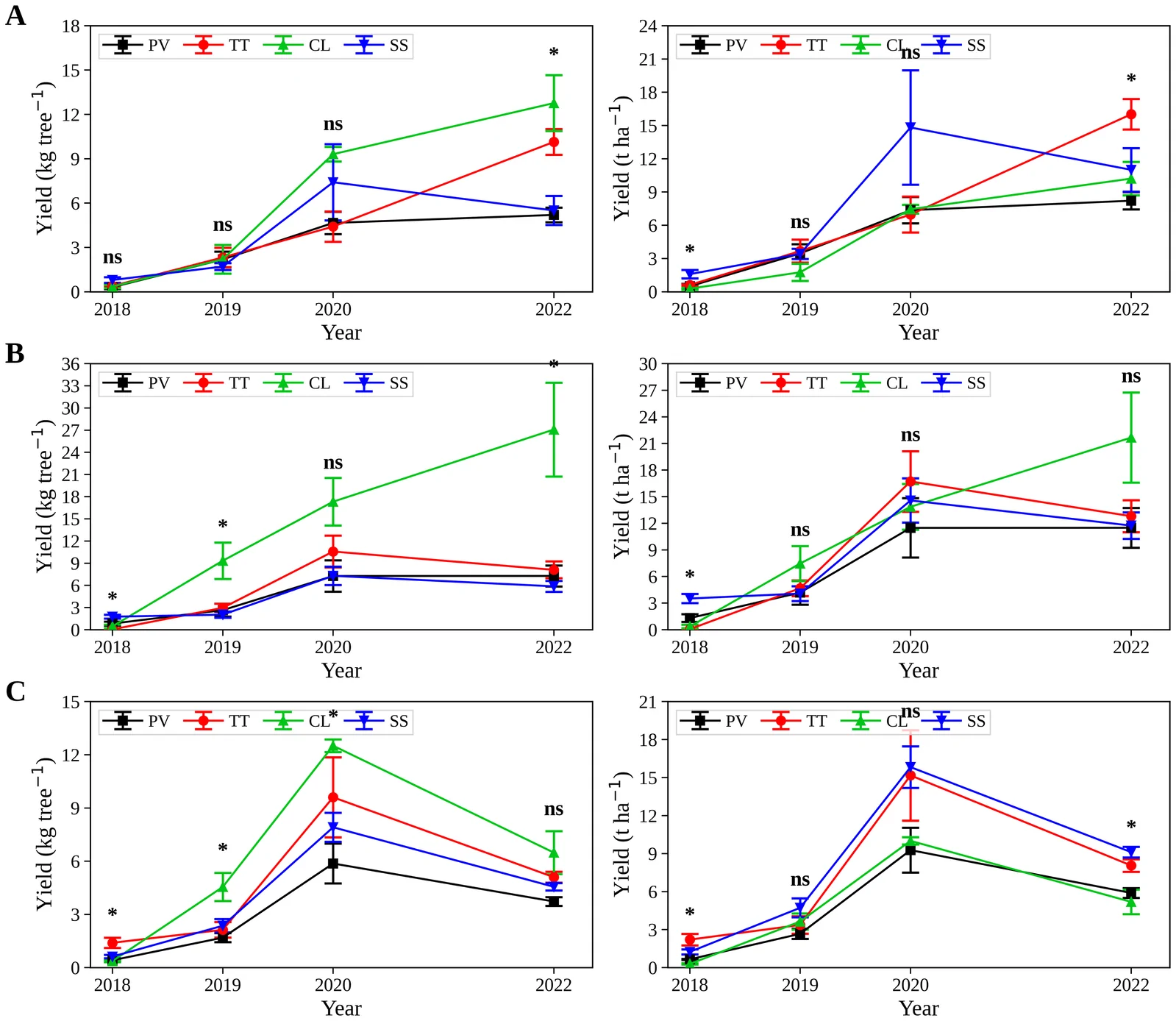
Yield (kg tree^−1^) and yield (t ha^−1^) of Extreme^®^ 314 (A), Extreme^®^ 436 (B), and Extreme^®^ 568 (C) peach cultivars trained to different training systems. No crop was recorded in 2021 due to severe frost. PV, Perpendicular-V; TT, Tatura Trellis; CL, Central Leader; SS, Slender Spindle. Values are presented as mean ±SE (*n* = 4). * indicates a significant training-system effect at *P* ≤ 0.05; ns indicates a non-significant effect.

Cumulative yield was significantly affected by training system, with significant training system × cultivar interactions detected for both yield per tree and yield per hectare (Table S1). The highest cumulative yield per hectare was obtained with SS in Extreme^®^ 314 (30.8 t ha^−1^), CL in Extreme^®^ 436 (43.4 ± 6.1 t ha^−1^), and SS in Extreme^®^ 568 (30.9 t ha^−1^) (Table 2). CL produced the highest cumulative yield per tree in all three cultivars, including 54.2 ± 7.6 kg tree^−1^ in Extreme^®^ 436. Yield efficiency and crop load also showed significant training system × cultivar interactions (Table S1). Yield-efficiency responses therefore varied among cultivar × training-system combinations, while PV generally showed comparatively low values. CL showed high crop load and yield efficiency in Extreme^®^ 436 and Extreme^®^ 568, whereas Extreme^®^ 314 responded differently (Table 2).

### Fruit characteristics

Fruit weight was significantly affected by cultivar and by the training system × cultivar interaction, whereas the main effect of training system was not significant (Table S1). The late-season cultivar Extreme^®^ 568 (234.7 g) produced the largest fruits, followed by Extreme^®^ 436 (196.3 g), across all training systems. While fruit weight in Extreme^®^ 314 showed little response to training system, the largest fruits for Extreme^®^ 436 were obtained under SS (211.7 g) and PV (200.7 g), whereas for Extreme^®^ 568, the highest values were recorded under CL (247.2 g) and TT (240.1 g) (Table 2).

Flesh firmness showed a significant training system × cultivar interaction, whereas the main effect of training system was not significant (Table S1). The highest firmness values for Extreme^®^ 314 and Extreme^®^ 436 were obtained under TT and PV systems, while no clear system separation was evident in Extreme^®^ 568 (Table 2).

SSC and titratable acidity (TA) showed significant training system × cultivar interactions (Table S1). Training systems affected SSC most clearly in Extreme^®^ 314, with the highest values under SS (13.16° Brix) and TT (12.62° Brix), whereas TA patterns varied among cultivars (Table 2). Fruit skin color differed among cultivars, with the lowest hue values and darker red coloration observed in Extreme^®^ 314 (28.15°) and more yellowish tones in Extreme^®^ 568 (37.15°). The training system × cultivar interaction for skin hue was not significant (Table S1); therefore, within-cultivar numerical differences in hue are interpreted descriptively.

### Correlation analysis

Tree canopy volume (TCV) was positively correlated with cumulative yield per tree in all cultivars (*r* = 0.66, 0.88, and 0.66 for Extreme^®^ 314, 436, and 568, respectively), but its relationship with cumulative yield per hectare differed among cultivars: negative in Extreme^®^ 314 (*r* = −0.53) and Extreme^®^ 568 (*r* = −0.49) and positive in Extreme^®^ 436 (*r* = 0.56) (Fig. 4). TCV was positively correlated with summer pruning weight in all three cultivars (*r* = 0.61–0.71). Cumulative yield efficiency showed strong positive correlations with cumulative crop load across cultivars (*r* = 0.88–0.99). Labor efficiency was negatively correlated with TCV in Extreme^®^ 314 (*r* = −0.55) and Extreme^®^ 568 (*r* = −0.57), but not in Extreme^®^ 436 (*r* = 0.26).

**Figure 4:**
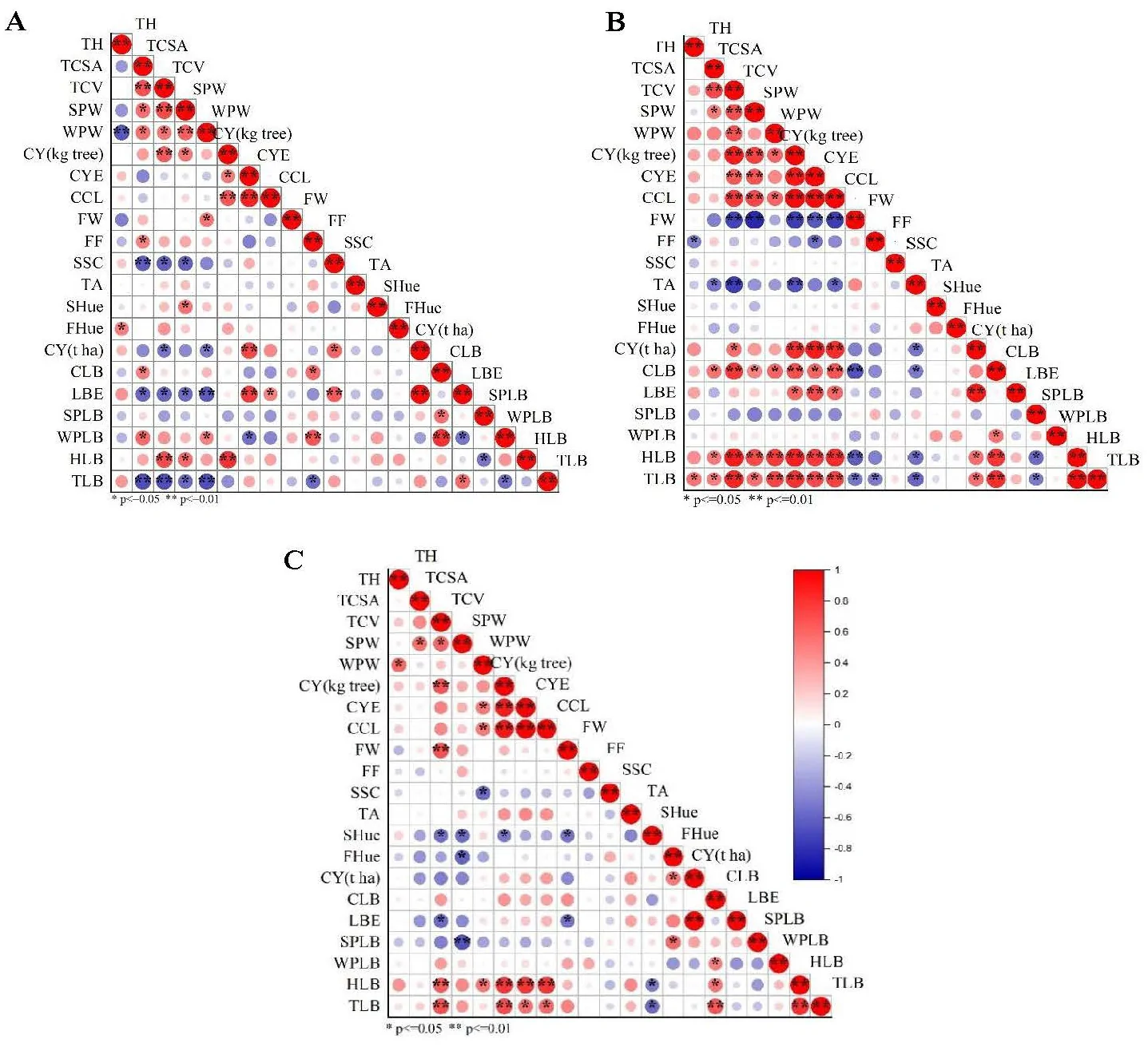
Pearson correlation matrices among growth, yield, fruit quality, and labor parameters. (A) Extreme^®^ 314; (B) Extreme^®^ 436; (C) Extreme^®^ 568. Self-correlations are shown in dark red. Color indicates the direction (positive or negative) of the correlation, while circle size represents the strength of the correlation. TH, tree height; TCSA, trunk cross-sectional area; TCV, tree canopy volume; SPW, summer pruning weight; WPW, winter pruning weight; CY (kg tree^−1^), cumulative yield per tree; CYE, cumulative yield efficiency; CCL, cumulative crop load; FW, fruit weight; FF, fruit firmness; SSC, soluble solids content; TA, titratable acidity; SHue, skin hue value; FHue, flesh hue value; CY (t ha^−1^), cumulative yield per hectare; CLB, cumulative labor; LBE, labor efficiency; SPLB, summer pruning labor; WPLB, winter pruning labor; HLB, harvest labor; TLB, thinning labor. * and ** indicate significant Pearson correlations at *P* < 0.05 and *P* < 0.01, respectively. Correlations were calculated separately for each cultivar using plot-level observations (*n* = 16 per cultivar) and are interpreted as exploratory.

Fruit weight (FW) showed cultivar-dependent relationships with canopy size. FW was essentially unrelated to TCV in Extreme^®^ 314 (*r* = 0.03), strongly negatively correlated in Extreme^®^ 436 (*r* = −0.73), and positively correlated in Extreme^®^ 568 (*r* = 0.68). In Extreme^®^ 436, FW was also negatively correlated with cumulative crop load (*r* = −0.74). Harvest labor was positively associated with TCV in all three cultivars (*r* = 0.65–0.89). Because these correlations retain the experimental treatment and block structure, they are interpreted as exploratory associations rather than independent confirmatory tests.

### Hierarchical clustering heatmap and PCA

Heatmap analysis revealed cultivar-specific clustering patterns among training systems. The dendrogram order was SS–CL–PV–TT for Extreme^®^ 314, CL–SS–PV–TT for Extreme^®^ 436, and CL–PV–TT–SS for Extreme^®^ 568 (Figs. 5A–5C). Thus, similarity among training systems differed among cultivars rather than showing a common clustering pattern.

**Figure 5:**
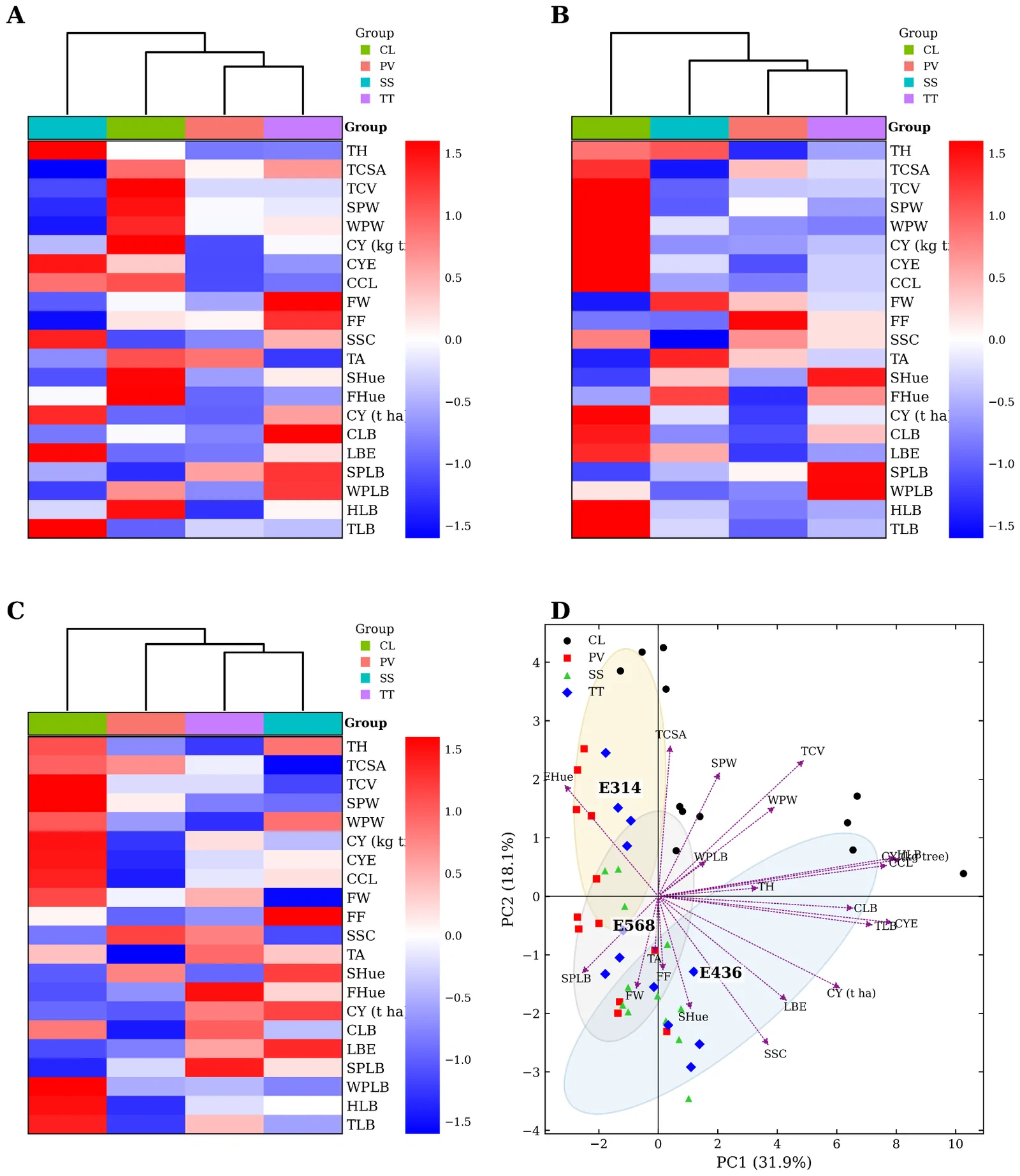
Hierarchical clustering heatmaps of Extreme^®^ 314 (A), Extreme^®^ 436 (B), and Extreme^®^ 568 (C) and principal component analysis of all measured parameters (D). Heatmap values are shown using a false-color scale, with red and blue indicating relative increases and decreases, respectively. Heatmaps were constructed from 12 cultivar × training-system treatment means, whereas PCA was performed using 48 plot-level observations after standardization. Both analyses are interpreted as exploratory. All abbreviations are defined in Fig. 4.

PCA supported a cultivar-dependent multivariate response. The first two components explained 31.9% (PC1) and 18.1% (PC2) of the total variance, respectively (49.9% cumulative; Fig. 5D). Treatments with larger canopies, greater pruning requirements, and higher per-tree productivity tended to separate from more compact and labor-efficient combinations, with Extreme^®^ 436 under CL particularly associated with high cumulative yield, crop load, and harvest labor. Because the first two components represented approximately half of the total variance, the PCA is interpreted as an exploratory summary rather than confirmatory evidence of treatment separation.

The cultivar × training-system combinations occupied different regions of the PCA space. Extreme^®^ 436 under CL was clearly separated toward the positive side of PC1 and was also positioned on the positive side of PC2, consistent with its comparatively high cumulative yield, crop load, and harvest-labor values. In Extreme^®^ 314, CL, PV, and TT were generally positioned on the negative side of PC1 and the positive side of PC2, with CL showing the strongest positive displacement along PC2, consistent with a greater contribution of vegetative-growth traits. In Extreme^®^ 436, TT and SS occupied the positive-PC1/negative-PC2 region, whereas PV was positioned on the negative side of both components. In Extreme^®^ 568, PV, SS, and TT were predominantly distributed in the negative-PC1/negative-PC2 region, whereas CL was positioned in the positive region of both components. Despite these general tendencies, considerable overlap among individual plot-level observations was evident. Therefore, the PCA and clustering analyses were interpreted as exploratory descriptions of multivariate patterns rather than as inferential tests of cultivar × training-system interaction.

## Discussion

### Phenological evaluation

The longer flowering period of Extreme^®^ 314 trained to TT and PV systems is consistent with previous studies indicating that more open or planar canopy architectures can slightly delay flowering and ripening by modifying light interception and canopy microclimate, although phenological responses were mainly cultivar-dependent (Nuzzo, Dichio & Xiloyannis, 2002; Robinson, Andersen & Hoying, 2006; Uberti et al., 2019).

The earlier ripening and shorter harvest period observed under SS and CL systems may be associated with increased light exposure and a more upright canopy structure, which can accelerate fruit development. Similarly, Uberti et al. (2019) reported that ripening occurred 5 days earlier in the central leader system compared to the Y system. The shorter harvest period may offer practical benefits, improving labor efficiency and reducing harvest costs. However, the observed differences in flowering and harvest may also be influenced by the early bearing stage of the orchard, as the first productive years are often characterized by irregular floral distribution and ongoing canopy adjustment.

### Tree growth characteristics

The greater tree height observed under SS training may be attributed to the promotion of vertical growth along a single central axis, while the smaller canopy volume indicates that this vigor was concentrated within a narrow canopy. In contrast, CL produced greater TCSA and canopy volume, reflecting a broader canopy structure. These differences are consistent with the general influence of canopy architecture and planting density on tree size and orchard spatial efficiency reported for peach training systems (Anthony & Minas, 2021). This density-related pattern is also consistent with Robinson et al. (2012), who reported a negative relationship between planting density and peach tree size.

The higher TCSA and canopy volume observed under CL relative to SS across cultivars suggest that central-axis architecture can support greater overall vegetative development. CL also produced the greatest cumulative summer-pruning weight within each cultivar, consistent with its larger canopy. However, the training system × cultivar interaction was not significant for summer-pruning weight, so this response should not be interpreted as cultivar-specific.

These findings agree with Rufato et al. (2004), who reported that the CL system generates denser canopies with greater foliage volume than Y-shaped systems. Similarly, Loreti & Massai (2002) emphasized the greater need for summer pruning in CL systems, particularly under fertile soil conditions and when using vigorous rootstocks, while noting that SS systems generally require less pruning. In the SS system, the increased winter pruning weight observed in Extreme^®^ 568 may be related to the formation and subsequent removal of thick structural branches during the early years of training. The PV and TT systems showed similar summer pruning requirements across cultivars and required more summer pruning than SS, second only to CL. In both systems, scaffold branches inclined more than 40–45° from the vertical frequently stimulated upright shoot growth within the canopy, increasing the need for summer pruning.

### Labor assessment

The pruning labor results indicate that canopy architecture influenced management requirements, but the interpretation depends on the unit of expression (Glenn et al., 2011; Taylor, 2003; Iglesias, Reighard & Lang, 2023). On a per-hectare basis, summer-pruning labor was greatest overall in TT, while winter-pruning labor was similarly high in TT and CL; the training system × cultivar interactions for these two pruning-labor variables were not significant. This differs from thinning and harvesting, for which significant interactions indicated that the magnitude of system differences depended on cultivar. The high harvest requirement of CL is consistent with its larger canopy and high per-tree crop, whereas PV consistently had the lowest harvest labor per hectare. The labor advantage observed for PV is consistent with the management rationale of perpendicular-V/Y-shaped peach systems, which were developed in part to simplify cultural operations and improve labor-use efficiency (DeJong et al., 1994; Anthony & Minas, 2021).

Beyond pruning, training system architecture also influenced thinning and harvesting labor by determining canopy accessibility and crop distribution. Although PV and TT share a dual-leader architecture, their labor responses were not identical. Differences in canopy geometry, scaffold orientation, planting density, and the trellis-supported structure of TT may contribute to these contrasting management requirements (Iglesias, Reighard & Lang, 2023; Zhang et al., 2023); however, these architectural components were not evaluated independently in the present study, and their individual contributions cannot be separated.

When yield was evaluated relative to labor inputs, SS showed the highest overall mean labor efficiency, consistent with previous peach training-system research on labor efficiency (Oran et al., 2024) and with the management advantages reported for compact/high-density canopy systems (Sansavini & Corelli-Grappadelli, 1997; Sobierajski et al., 2019). However, labor efficiency itself did not show a significant training system × cultivar interaction, whereas cumulative labor, thinning labor, and harvest labor did. Thus, PV can reduce operation-specific labor without necessarily maximizing yield per labor hour, whereas TT represents a different trade-off: it produced numerically higher cumulative yield per hectare than PV in all three cultivars but also required greater cumulative labor. Establishment costs were not evaluated and should be considered separately, particularly for trellis-supported systems.

### Yield, cumulative yield, and yield efficiency

Early and cumulative yield responses were cultivar-dependent, consistent with the significant training system × cultivar interactions for cumulative yield per tree and per hectare. CL supported high per-tree production, consistent with its larger canopy and greater number of potential fruiting sites (Rufato et al., 2004), but hectare-based productivity also reflected planting density. This distinction was especially clear for Extreme^®^ 436, for which CL achieved the highest cumulative yield per hectare, whereas the highest hectare-based yields for Extreme^®^ 314 and Extreme^®^ 568 were obtained with SS.

Nevertheless, system comparisons should be interpreted alongside planting density effects. Increased density can reduce yield per tree while increasing land-use productivity (Robinson, Andersen & Hoying, 2006; Reig et al., 2019; Corelli-Grappadelli & Marini, 2008; Lauri & Corelli-Grappadelli, 2014). The present results similarly show that system rankings depend on cultivar and on whether productivity is expressed per tree or per hectare. Contrasting rankings of central-axis and Y/V-type peach systems have also been reported in the literature, emphasizing the importance of planting density, crop load, cultivar, and orchard design when comparing architectures (Anthony & Minas, 2021). Comparable effects of training system and planting distance on peach productivity have also been reported by Çetinbaş et al. (2021).

Seasonal variation also highlighted the influence of frost on orchard productivity. The severe frost in 2021 prevented a representative crop, so cumulative yield represents performance across the harvested seasons rather than uninterrupted annual production throughout 2018–2022. Because the present study was not designed to compare frost tolerance among training systems, no system-specific frost mechanism is inferred.

Among the dual-leader systems, cumulative yield per hectare was numerically higher under TT than PV in all three cultivars, although within-cultivar Tukey groupings overlapped. This pattern should therefore not be interpreted as a universal significant advantage of TT. Earlier evaluations of Perpendicular-V systems likewise emphasized that system value should be judged not only by yield but also by orchard management and the efficiency of cultural operations (DeJong et al., 1994). Thus, the lower harvest labor observed for PV represents a relevant management advantage even though PV did not maximize cumulative yield per hectare.

In contrast to Lal, Sharma & Singh (2017), the Central Leader system did not show uniformly low yield efficiency in the present study, indicating that its performance was cultivar-dependent. CL produced particularly high crop load and yield efficiency in Extreme^®^ 436 and also high values in Extreme^®^ 568, whereas Extreme^®^ 314 responded differently. These results reinforce the need to interpret yield efficiency and crop load at the cultivar × training-system level rather than from training-system main effects alone.

This outcome may appear inconsistent with structural limitations often attributed to central-axis designs. Hoying, Robinson & Anderson (2007) noted that upright peach growth can increase vertical scaffold development and reduce canopy light distribution, while Y-type systems have been associated with improved canopy spatial distribution (Caruso et al., 1998). In the present study, however, within-canopy light distribution and cultivar-specific branch architecture were not measured directly, so the strong Extreme^®^ 436 response under CL cannot be assigned to a single canopy mechanism. Differences in planting distance and orchard geometry may also contribute to system performance (Trentacoste, Connor & Gómez-del Campo, 2015).

### Fruit characteristics

Training-system effects on fruit weight were cultivar-dependent. Extreme^®^ 314 showed little response, whereas Extreme^®^ 436 produced larger fruit under SS and PV and Extreme^®^ 568 under CL and TT. Similar variability has been reported in peach, with fruit-size responses depending on cultivar, crop load, planting density, resource availability, and management conditions (Grossman & DeJong, 1995; Caruso, Barone & Di Vaio, 2001; Farina, Bianco & Inglese, 2005; Robinson, Andersen & Hoying, 2006; Pasa et al., 2017; Reig et al., 2019).

Regarding firmness, TT and PV had relatively high values in Extreme^®^ 314 and Extreme^®^ 436, but this pattern was not evident in Extreme^®^ 568. Fruit firmness can be influenced by harvest maturity, crop load, and ripening conditions (Uberti et al., 2019; Vicente et al., 2007; Christofi et al., 2021); however, the present data do not identify a single mechanism. The result is therefore better interpreted as a cultivar-specific response than as a general firmness advantage of dual-leader systems.

Differences in SSC and titratable acidity among systems were also cultivar-dependent, consistent with the significant interactions. Previous studies have reported variable effects of training system on these traits, reflecting the combined influence of genotype, crop load, light environment, and source–sink balance (Crisosto & Crisosto, 2005; Lal, Sharma & Singh, 2017; Bussi et al., 2015; Pasa et al., 2017). The higher SSC under SS and TT in Extreme^®^ 314 was not reproduced consistently in the other cultivars, and TA patterns likewise differed among cultivars.

Training-system effects on skin color should be interpreted cautiously. The factorial analysis did not detect a significant training system × cultivar interaction for skin hue (Table S1). Although treatment means in Extreme^®^ 568 showed a wider numerical range, this pattern is descriptive rather than evidence of a cultivar-specific system effect. Canopy light distribution can influence peach skin pigmentation (Caruso, Barone & Di Vaio, 2001; Robinson, Andersen & Hoying, 2006), but within-canopy irradiance was not measured in the present study.

### Correlation analysis

The correlation analysis emphasized cultivar-specific rather than universal relationships. Larger canopy volume was associated with greater cumulative yield per tree in all cultivars, but its relationship with cumulative yield per hectare was negative in Extreme^®^ 314 and Extreme^®^ 568 and positive in Extreme^®^ 436. This contrast is consistent with the fact that hectare-based productivity combines individual-tree performance with planting density and cultivar response. Positive associations between canopy volume and pruning weight further indicate that vigorous canopies require greater canopy-management inputs (Wünsche & Lakso, 2000).

Labor- and fruit-related correlations were likewise cultivar-dependent. Harvest labor was positively associated with canopy volume across cultivars, consistent with greater management demand in larger trees (Minas, Tanou & Molassiotis, 2018; Anthony & Minas, 2021). The relationship between fruit weight and canopy volume ranged from essentially zero in Extreme^®^ 314 to strongly negative in Extreme^®^ 436 and positive in Extreme^®^ 568. The negative fruit-weight–crop-load relationship in Extreme^®^ 436 is consistent with the well-established trade-off between fruit size and fruiting intensity (Grossman & DeJong, 1998). Because the correlation matrices retain the experimental treatment structure, these associations are interpreted as exploratory rather than causal.

The heatmap and PCA provide an exploratory synthesis of these cultivar-dependent responses. The different dendrogram orders among cultivars and the 49.9% cumulative variance explained by PC1 and PC2 indicate that the multivariate patterns should be viewed as supportive, descriptive evidence rather than as independent statistical proof of treatment separation.

## Conclusion

This study compared single- and dual-leader peach training systems in terms of tree architecture, yield performance, fruit quality, and labor requirements across three cultivars. Phenological differences among systems were limited, with slightly earlier and more compact ripening observed in SS and CL. Regarding tree architecture, SS maintained smaller canopy volumes, whereas CL generally promoted larger canopies and greater pruning requirements.

Labor responses varied according to both operation and cultivar. PV consistently had the lowest harvest labor per hectare, whereas SS showed the highest overall labor efficiency. Yield responses also depended on cultivar and planting configuration. Cumulative yield per hectare was highest under SS for Extreme^®^ 314, CL for Extreme^®^ 436, and SS for Extreme^®^ 568. These findings indicate that the relative advantage of a training system cannot be generalized across cultivars solely on the basis of planting density.

Overall, the four training systems presented different yield–labor trade-offs. TT produced numerically higher cumulative yield per hectare than PV in all three cultivars but required greater cumulative labor, whereas PV provided a clear harvest-labor advantage. CL may remain advantageous for particular cultivars, as demonstrated by the highest cumulative yield per hectare obtained for Extreme^®^ 436, while SS combined compact canopies with high overall labor efficiency and high hectare-based yield in Extreme^®^ 314 and Extreme^®^ 568. Therefore, selection of a training system should consider cultivar characteristics together with planting density, productivity, fruit quality, and labor requirements rather than assuming a uniform system response across cultivars. Further long-term and multi-site studies are needed to evaluate the stability and sustainability of these responses.

## Supplemental Information

10.7717/peerj.21816/supp-1 Supplemental Information 1 Data and statistical analysis outputs for growth, yield, fruit quality, and labor traits in peach cultivars under different training systems The verified plot-level data and supporting analytical datasets used in this study. Each plot-level observation represents the mean of five trees within one experimental plot in a randomized complete block design with four blocks. Includes plot-level data for growth, yield, fruit quality, and labor traits, annual yield data, the plot-level dataset used for PCA (*n* = 48), treatment means used for the heatmap (*n* = 12), and ANOVA effect-test summaries (df, *F*, and *P* values) in Supplementary Table S1. A data dictionary provide details on the dataset structure, abbreviations, and units.

## Additional Information and Declarations

### Data Availability

The following information was supplied regarding data availability: The raw data is available in the Supplemental File.
